# Conservation and Variability of Synaptonemal Complex Proteins in Phylogenesis of Eukaryotes

**DOI:** 10.1155/2014/856230

**Published:** 2014-07-23

**Authors:** Tatiana M. Grishaeva, Yuri F. Bogdanov

**Affiliations:** Vavilov Institute of General Genetics, Russian Academy of Sciences, Gubkin Street 3, GSP-1 Russian Federation, Moscow 119991, Russia

## Abstract

The problems of the origin and evolution of meiosis include the enigmatic variability of the synaptonemal complexes (SCs) which, being morphology similar, consist of different proteins in different eukaryotic phyla. Using bioinformatics methods, we monitored all available eukaryotic proteomes to find proteins similar to known SC proteins of model organisms. We found proteins similar to SC lateral element (LE) proteins and possessing the HORMA domain in the majority of the eukaryotic taxa and assume them the most ancient among all SC proteins. Vertebrate LE proteins SYCP2, SYCP3, and SC65 proved to have related proteins in many invertebrate taxa. Proteins of SC central space are most evolutionarily variable. It means that different protein-protein interactions can exist to connect LEs. Proteins similar to the known SC proteins were not found in Euglenophyta, Chrysophyta, Charophyta, Xanthophyta, Dinoflagellata, and primitive Coelomata. We conclude that different proteins whose common feature is the presence of domains with a certain conformation are involved in the formation of the SC in different eukaryotic phyla. This permits a targeted search for orthologs of the SC proteins using phylogenetic trees. Here we consider example of phylogenetic trees for protozoans, fungi, algae, mosses, and flowering plants.

## 1. Introduction

Meiosis is a division of germ-line cells that involves recombination of genetic material and segregation of homologous chromosomes, leading to production of haploid gametes from a diploid cell, while mitosis preserves the initial chromosome number in both daughter cells. Meiosis is an obligatory component in sexual process in eukaryotes. The origin and evolution of the mechanism of meiosis and proteins involved in meiotic processes are a matter of discussion [1–6].

A principal difference between the results of meiosis and mitosis is determined by their difference in genetic control, chromosome structure, and chromosome behavior. A difference at the ultrastructural level appears as formation of meiosis-specific synaptonemal complexes (SCs), the ultrastructures that join homologous chromosomes into bivalents during pachytene stage of meiotic prophase I in the vast majority of eukaryotes. SC is necessary for specific organization of prophase meiotic chromosomes [7–9], synapsis of homologous chromosomes [7, 10, 11], and chiasma number per one SC sufficient for regular homologues segregation [8, 12].

SCs are formed of meiosis-specific proteins [11, 13, 14]. General organization of the SC is more or less similar among all eukaryotes examined in this respect, while ultrastructure of its morphological components slightly varies [8, 15]. In addition to the ultrastructural variation, low, if any, similarity was found between the specific proteins that build up SCs in plants, fungi, and animals [11, 14, 16]. It means that the functional conservation of the proteins as the material for constructing SCs is not associated with homology of their amino acid sequences. Thus, the general picture can presumably be presented as follows. Nonhomologous proteins build up the SCs, which are rather conserved ultrastructures of meiocytes and perform a common function in the course of meiosis in eukaryotic organisms [4]. It is still an enigma as to how very dissimilar proteins can build intracellular structures with principally similar morphology and function.

It is worthwhile to consider several details of SC protein diversity. Lateral elements (LEs) of the SC are formed on the basis of chromosomal axial elements, which connect sister chromatids and consist mostly of cohesins [17]. The LEs are joined together to produce the integral SC structure via a zipper of transversal filaments, which pass through the SC central space. Heads of the transversal filaments overlap in the middle of the central space to form a SC central element (CE) [4, 13].

Different meiosis-specific proteins of SCs are synthesized in generative cells on the eve or in the course of early stages of meiosis [17, 18]. Since the first SC proteins have been identified, yeast Hop1 and Zip1 [19–21], rodent SYCP1 [22, 23], SYCP2 [24], and SYCP3 [23, 25], the SC proteins that would be universal for all eukaryotes are sought by bioinformatics methods. However, it has been observed that the mammalian SC central space protein SYCP1 is nonhomologous to yeast SC central space protein Zip1 [26]. Their functional analog in plants, ZYP1 from* Arabidopsis*, has only 20% identity with two former proteins [27, 28]. The same is true for the* Drosophila* protein encoded by gene* c(3)G* [29, 30] and for nematode SYP-1 [31]. A secondary structure of some parts of polypeptide chains of all these proteins is only their common feature; that is, all they have globular domains at the N and C ends and a central *α*-helical domain. The long *α*-helix (coiled coil) makes the molecule rod shaped [10, 32, 33], which is essential for producing transversal filaments in the SC central space. Murine low-molecular-weight proteins that modify the structure of the SC central space (SYCE1, SYCE2, SYCE3, and TEX12) initially were considered as having only vertebrate orthologs [34–36].

The mammalian LE proteins SYCP2 and SYCP3 have only a low similarity to their counterparts of yeast (Hop1, Red1), nematode (HIM-3), and* Arabidopsis* (ASY1), identified more recently [23, 37–40]. A HORMA domain is the only feature common for certain proteins of the SC LEs. Thus, the SCs are similar in general morphology (structural plan) but differ in ultrastructure and consist of different proteins in yeast, nematode,* Drosophila*, mammals, and* Arabidopsis*. These organisms are hereafter referred to as models to study the SC proteins.

Ramesh et al. [3] have carried out an interesting study, searching for orthologs of key meiotic proteins in the proteomes of Archaea, bacteria, and 15 eukaryotes of different taxa from protists to human. Of all structural meiotic proteins, only the LE component Hop1 was included in the analysis. Hop1 orthologs were found in almost all of the species examined, including human and mouse. The mouse protein was identified as HORMAD1, which is considered below.

Fraune et al. [41] made the next step and found SYCP1 and SYCP3 orthologs in the proteomes of various metazoan taxa, including Placozoa, Porifera, and Coelenterata. Protein fragments identified as the most conserved, rather than total amino acid sequences, were used as queries. A bioinformatics search was supplemented by an experimental verification in the case of* Hydra*. In a recent study, Fraune et al. [42] extended their experiments and traced the origin of proteins structuring the SC central space. They found that SYCE2 and TEX12 are conserved in Metazoa, SYCE1 appears in Bilateria, and SYCE3 is specific for Vertebrata.

Our hypothesis is that the homology of some polypeptide domains rather than that of whole proteins is critical in constructing ultrastructural components of SCs in remote taxa. The main objective of our work was to search the proteomes of diverse eukaryotic taxa, especially those not yet examined before like different unicellular animals, algae, lower fungi, mosses, and some others, for proteins and their domains similar to the SC proteins of the model organisms. We are the first to consider almost all known SC proteins of the model organisms when seeking related proteins in the proteomes of main eukaryotic taxa in one study. We obtained a large list of proteins, which can serve as a potential source for a targeted search for orthologs of the SC proteins using phylogenetic trees and constructed example trees for groups of organisms so far poorly studied in this respect.

## 2. Materials and Methods

In total, approximately 11 million proteins from approximately 5000 proteomes of all main eukaryotic groups were tested. The taxonomy available from the NCBI database (http://www.ncbi.nlm.nih.gov/) was used. The SC proteins of the seven model eukaryotic species, namely, yeasts* Schizosaccharomyces pombe *and* Saccharomyces cerevisiae, *plant* Arabidopsis thaliana,* nematode* Caenorhabditis elegans*, insect* Drosophila melanogaster, *fish* Danio rerio, *andmammal* Mus musculus* (as most common objects in meiosis studies) were used as queries in comparisons with the above proteins (Tables 1 and 2). In these model organisms, SC proteins had been isolated and studied experimentally, except* D. rerio* whose genome was well studied and SC proteins were revealed with bioinformatic methods. In one experiment,* D. rerio *was substituted by another fish* Anoplopoma fimbria. *This is why we did not include Af into list of main model organisms. As soon as the mammalian SC proteins SYCE and TEX, discovered recently, were found in mouse, we consider the mouse as the most representative mammalian species, if only one species is to be chosen as query in laborious computer database monitoring. Human SC proteins are very similar to their mouse counterparts, and their use as queries will add no new results, comparatively to mouse.

The amino acid sequences of SC proteins were sought in the NCBI (http://www.ncbi.nlm.nih.gov/) and UniProtKB/Swiss-Prot (http://www.uniprot.org/uniprot/) databases. The functional domains of the above proteins were identified using CDART software (http://www.ncbi.nlm.nih.gov/Structure/cdd/wrpsb.cgi?). Random amino acid sequences were generated on the basis of native proteins by the RandSeq program (http://au.expasy.org/tools/randseq.htm) to serve as a control in estimating protein similarity.

Proteins similar to SC proteins were sought in the proteomes of main eukaryotic groups using NCBI Protein BLAST software (http://www.ncbi.nlm.nih.gov/blast/Blast.cgi?PROGRAM=blastp&BLAST_PROGRAMS=blastp&PAGE_TYPE=BlastSearch&SHOW_DEFAULTS=on&LINK_LOC=blasthome#). The taxa examined are summarized in Tables 3–6. The taxa with only few protein sequences available from the databases were pooled. The parameters of our PROTEIN BLAST search were as follows. Maximum target sequences are 1000 or 5000 in different studies (maximum number of aligned sequences to display; the actual number of alignments may be smaller or greater than this). Expect threshold is 100 (this setting specifies the statistical significance threshold for reporting matches against database sequences. The default value (100) means that 100 such matches are expected to be found merely by chance). The others were default parameters. The similarity index score (BLAST output) is based on three parameters: the number of matching amino acid residues, the number of amino acid residues of the same type, and the number of gaps, that is, cases where a certain position is occupied by an amino acid residue in one protein and is empty in another. For each of the SC proteins, the scores for similarity with proteins of the proteomes of a particular eukaryotic group were compared for the protein in question and its “random” analog. The significance of the similarity index was characterized by the* E*-value, which reflects the number of similar proteins that might be selected at random by the BLAST program and is calculated by the BLAST itself. Maximal scores, found by BLAST, in their increment order, are summarized in the tables (see Results and Discussion). The SC proteins with a score lower than 50 are not listed because their similarity was considered to be very low. When similar scores were obtained for a native and a “random” protein, we compared their scores averaged over the 10 best search results. Comparisons by Student's* t*-test were performed using STATISTICA software v.7 (http://www.statsoft.com).

As the score depends on the sequence length, it is incorrect to compare the scores obtained for differently sized proteins. However, a “vertical” score comparison, that is, a comparison of scores obtained for similar proteins from different eukaryotic groups, seems proper. It is also clear that absolute score values are of little importance when the scores are low and comparable with those of random sequences.

Phylogenetic trees were constructed using the constraint-based multiple protein alignment tool (COBALT) from the NCBI package (http://www.ncbi.nlm.nih.gov/tools/cobalt/cobalt.cgi?CMD=Web). Default parameters were used in multiple sequence alignment. At the final stage of constructing trees, the fast minimum evolution algorithm was employed. Protein IDs are listed in figure captions. Proteins with the highest similarity to Hop1 and ASY1 were considered for each taxon.

## 3. Results and Discussion

A list of SC proteins of seven model organisms is provided in Table 1. Protein size and some characteristics of their functional domains are shown in Table 2. Similar proteins were sought in proteomes of all eukaryotic species available in databases at the time of study (see Materials and Methods) using the BLAST program. The degree of similarity was estimated as score index (see Materials and Methods).

### 3.1. Regularities of Distribution of the Studied Proteins among Eukaryotes

As expected, extremely high scores were obtained when comparing yeast SC proteins with yeast proteomes, nematode SC proteins with nematode proteomes, and so forth (i.e., a protein with a cognate proteome). The result testified that the method worked; obviously, these data were not included in the tables. The scores were depending on the protein size. For instance, a score of 178 was obtained for mouse SYCE3 (88 residues), while mouse SYCP2 (1500 residues) had a score of 3106.

#### 3.1.1. Algae, Mosses, Flowering Plants, and Fungi

Proteins similar to the yeast and* Arabidopsis* SC proteins Hop1, ASY1, and ASY2 were found in the proteomes of algae, mosses, fungi, and higher plants (Table 3). The highest similarity to the vertebrate SYCP1 proteins was observed for proteins from the proteomes of green algae, brown algae, and ascomycetes (Table 3), which seems to be due to their secondary structure similarity (see below). All of the unicellular eukaryotic groups examined had proteins more or less similar to the SC proteins of the model organisms (Table 4). The highest scores were obtained for the group Fornicata-Parabasalia-Heterolobosea. Their Hop1 orthologs are already annotated in databases. Parabasalia is possibly the most ancient eukaryotic group with sexual reproduction [44].

**Figure 1:**
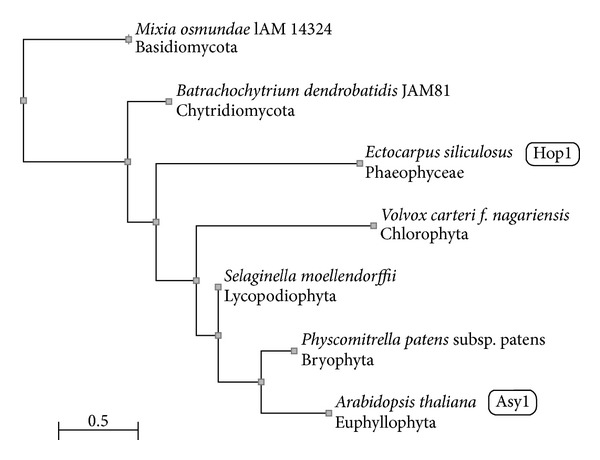
Phylogenetic tree of algae, fungi, mosses, and green plants based on proteins similar to Hop1/ASY1 in proteomes of these organisms found with the use of maximal scores. The initial set of proteins used to construct the* fast minimum evolution* tree included RefSeq: XP_002957345 (*Volvox carteri*, Chlorophyta); GenBank: CBN75586, annotated as Hop1 homolog (*Ectocarpus siliculosus*, Phaeophyceae); RefSeq: XP_002995702 (*Nosema ceranae*, Microsporidia); GenBank: EGF80506 (*Batrachochytrium dendrobatidis*, Chytridiomycota); RefSeq: NP_012193.1, SC protein Hop1 (*Saccharomyces cerevisiae*, Ascomycota); GenBank: GAA98305 (*Mixia osmundae*, Basidiomycota); RefSeq: XP_001760173 (*Physcomitrella patens*, Bryophyta); RefSeq: XP_002969766 (*Selaginella moellendorffii*, Lycopodiophyta); RefSeq: NP_564896.1, SC protein ASY1 (*Arabidopsis thaliana*, Euphyllophyta). The archaeal protein RefSeq: YP_003707339.1 (*Methanococcus voltae*, Archaea) was taken as control. Only species and higher taxa are indicated on the tree. Three proteins (from Archaea, Microsporidia, and Ascomycota) were automatically removed from the final version of the tree. The evolutionary distance between two sequences was modeled as expected fraction of amino acid substitutions per site given the fraction of mismatched amino acids in the aligned region (according to [43]).

As an example of practical usage of found similarities, we constructed phylogenetic trees of HORMA-domain proteins similar to Hop1 of* Saccharomyces cerevisiae *and ASY1 of* Arabidopsis thaliana*. One species whose protein showed the highest similarity to Hop1 and ASY1 was selected from each of the taxonomic groups shown in Tables 3 and 4, with the exception of Ciliophora. The proteins were mostly counterparts. The protein IDs and source species are listed in figure captions. As control, we used a distantly related protein of the archaean* Methanococcus voltae* that displayed a low but still significant similarity to Hop1. One tree (Figure 1) was constructed for algae, fungi, mosses, and green plants. As expected, the archaean protein was automatically excluded by the program. The protein found in lower fungi (*Nosema ceranae*, Microsporidia) was also excluded by the program, which might be expected as well. An unexpected finding was that Hop1 of the yeast* S. cerevisiae* was rather distant from all of the other proteins included in the tree.

**Figure 2:**
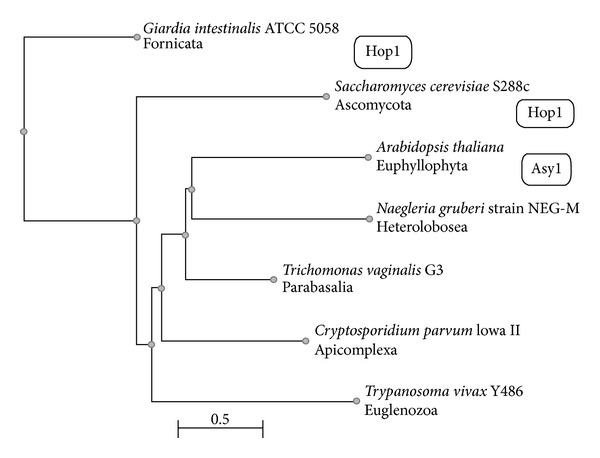
Phylogenetic tree of Hop1/ASY1-similar proteins found in the proteomes of unicellular eukaryotes. The initial set of proteins used to construct the* fast minimum evolution* tree included RefSeq: NP_012193.1, SC protein Hop1 (*Saccharomyces cerevisiae*, Ascomycota); RefSeq: NP_564896.1, SC protein ASY1 (*Arabidopsis thaliana*, Euphyllophyta); RefSeq: XP_001321336 (*Trichomonas vaginalis*, Parabasalia); RefSeq: XP_002675215 (*Naegleria gruberi*, Heterolobosea); GenBank: EET02094, annotated as Hop1 homolog (*Giardia intestinalis*, Fornicata); RefSeq: XP_626119 (*Cryptosporidium parvum*, Apicomplexa); GenBank: CCC51501 (*Trypanosoma vivax*, Euglenozoa); RefSeq: XP_001742099 (*Monosiga brevicollis*, Choanoflagellata). The archaeal protein RefSeq: YP_003707339.1 (*Methanococcus voltae*, Archaea) was taken as control. Only species and higher taxa are indicated on tree. Two proteins (from Archaea and Choanoflagellata) were automatically removed from the final version of the tree. The evolutionary distance between two sequences was modeled as expected fraction of amino acid substitutions per site given the fraction of mismatched amino acids in the aligned region (according to [43]).

Another tree (Figure 2) was constructed for proteins of unicellular eukaryotes and included the known Hop1 and ASY1 proteins. The* Methanococcus voltae* (Archaea) and* Monosiga brevicollis* (Choanoflagellata) proteins were excluded automatically, while clustering with* Giardia intestinalis* (Fornicata) Hop1 at the step of Cobalt tree construction. The Choanoflagellata proteins displayed, in fact, only a minor similarity to the SC proteins of the model organisms.

#### 3.1.2. Animals

Among the multicellular organisms listed in Table 5, mollusks had the highest scores of similarity between some of their proteins and the model SC proteins. SYCP2 and SYCP3 homologs of* Crassostrea gigas* (maximal scores 80 and 199, resp.) are annotated in the NCBI database. A SYCP3 homolog was additionally found in the proteomes of the sponge* Amphimedon queenslandica *(Score_max⁡_ = 116) and the coelenterate* Hydra magnipapillata *(Score_max⁡_ = 135). It should be noted that the sponge protein found in our search (Table 5) did not coincide with the SYCP3 described by Fraune et al. [41]. A protein annotated as SC65 occurred in the proteome of an ascarid nematode (Score_max⁡_ = 129). As a control, we compared SC65 for mouse and fish (score = 456) and SYCP3 for mouse and fish (score = 263). It is seen that the scores obtained for SC65 and SYCP3 similarities with proteins of the above eukaryotic groups were sufficiently high. All of the eukaryotic groups included in Table 5 had not only proteins similar to animal SC proteins, but also those similar to yeast Hop1 and plant ASY1.

To better understand the above values, it may also be helpful to consider the scores obtained for several other proteins. The highly conserved meiotic enzyme DMC1 showed the following maximal scores in comparisons of mouse DMC1 with proteins of other organisms: 622 for* Danio rerio*, 307 for* Caenorhabditis elegans*, 391 for* Arabidopsis thaliana*, 310 for* Drosophila melanogaster*, and 372 for* Saccharomyces cerevisiae* (our data). The maximal scores obtained in similar comparisons for the structural SC protein SYCP1, whose conservation is by far lower, were 320, 49, 38, 42, and 33, respectively. It is clear that scores that exceed 100 in comparisons of the SC proteins with coelenterate or mollusk proteomes may point to some relatedness of certain proteins, although their orthology is out of the question.

The most highly organized animals had proteins directly orthologous or highly similar to the SC proteins of the model organisms (Table 6). The hypothetical protein BRAFLDRAFT_118903 found by us in the proteome of* Branchiostoma floridae* (subtype Cephalochordata) is similar to different proteins forming the SC transversal filaments. The protein contains multiple Filamin-type immunoglobulin domains (Filamin/ABP280 repeats), and a distinct *α*-helix forms in the central part of its molecule (our data), as in SC transversal filament proteins of model organisms. The protein CBY10027.1 of Tunicata showed an additional similarity to a random analog of mouse SYCP1 (Table 6). The protein contains GCC2_GCC3 repeats and a Trichoplein domain (for more detail, see below) and similarly forms an *α*-helix, although it is in the C-terminal region of the molecule.

#### 3.1.3. Taxa without “Standard” SC Proteins

Several eukaryotic taxa were not found to have proteins with a considerable similarity to the SC proteins of the model species selected for our comparisons. We did not include these taxa in the tables and just list them here. These were Rhodophyta, Euglenophyta, Chrysophyta, Charophyta, Xanthophyta, and Dinoflagellata among algae. Among animals, there were Mesozoa, Gnathostomulida, Bryozoa, Cycliophora, Myzostomida, and Nemertea; also there are Rotifera, Nematomorpha, Scalidophora, Acanthocephala, Entoprocta, and Gastrotricha from Coelomata. Likewise, no proteins similar to SC proteins were found in Tardigrada and Onychophora (Protostomia) and in Hyperotreti, Hyperoartia, and Chondrichthyes (Chordata).

Proteins with significant similarity to only FKBP6 (peptidyl-prolyl cis-trans isomerase) were found for several taxa, which were also not listed in the tables. The taxa included Cryptophyta, Diatoms, and Pelagophyceae (algae) and Perkinsea, Oomycota, and Labyrinthulomycota (Labyrinthulida), the two last groups additionally having proteins similar to mouse SYCP1. Among animals, Rhizaria, Myxosporea, amoeboid protists, and Annelida also have proteins similar to mouse FKBP6.

We did not detect any proteins similar to structural meiotic proteins in these eukaryotic groups possibly because only few of their proteins are available in databases. The exceptions are Rhodophyta, Dinoflagellata, Chondrichthyes, Oomycota, Rhizaria, and Annelida. For each of these phyla more than 10 proteomes are annotated in databases. It means that SCs in these taxonomic groups, if exist at all, lack typical proteins of model SCs and could be built of noncanonical proteins.

#### 3.1.4. Meiosis without SC and with Nontypical SCs

Meiosis is thought to appear simultaneously with mitosis [5] or to originate from mitosis [6]. Most components of molecular machinery necessary for initiating homologous pairing (e.g., meiosis-specific cohesin Rec8 and others) might arise as early as at the time of origin of protoeukaryotes [6], while SC components are possibly of a more recent origin. Key meiotic proteins have been found in the protist* Giardia intestinalis*, although the organism presumably lacks meiosis [3]. Both Ramesh et al. [3] and Cavalier-Smith [1] have assumed that meiosis arose quite early in eukaryotic evolution. However, only Hop1 of all SC proteins has been included in their analysis.

We could not establish whether SCs form during meiosis in all of our subjects. Meiosis proceeds without SCs formation in lower fungi, such as* Schizosaccharomyces pombe *and* Aspergillus nidulans* (cited from [8]). Meiosis is absent in the ascomycete* Candida albicans* and present in* Candida lusitaniae*, but both of the species similarly lack key SC proteins resembling SC proteins of the model organisms [45]. At the same time, the SC forms in the ascomycete* Neurospora crassa* and basidiomycete* Coprinus cinereus* [46–48]. The SCs form also in* Eimeria tenella* (Apicomplexa) [49] but not in its distant relative* Tetrahymena thermophila* (Ciliophora), while residual SC-like structures are observed in the protist* Stylonychia* [50]. The brown alga* Ectocarpus siliculosus* has meiosis [51], but only its Hop1 is annotated in databases (Table 3). Red algae and diatoms form the SCs [46]. The proteomes of algae, mosses, green plants, and fungi listed in Table 3 include proteins similar to yeast and plant LE proteins, including Hop1, ASY1, and ASY2. Yet the algal, moss, and fungal proteomes were not found to have proteins similar to yeast Zip1 or* Arabidopsis* ZYP1, which form transversal filaments in the SC. We detected only the proteins that have a rather low similarity to vertebrate transversal filament proteins. These eukaryotic groups evolved independently of each other [51]. Their SCs might include still unidentified proteins with a secondary structure characteristic of SC transversal filaments.

Interestingly, proteins with a high similarity to any known SC protein were not detected in the proteomes of Choanoflagellata, which are thought to be the nearest relatives of Metazoa among all unicellular organisms [52]. This observation may indicate that the known SC proteins arose in more highly organized eukaryotes. It is possible that each of the independent evolutionary lineages of multicellular eukaryotes (red and brown algae, green plants, fungi, and animals) has two categories of meiosis-specific proteins: a common set of basic meiotic proteins, as may be suggested from the findings reported by Ramesh et al. [3], and, additionally, a lineage-specific set of structural proteins, including SC proteins. The hypothesis is supported by the fact that proteins similar to SYCP1 and SYCP3 are found in the proteomes of basic Metazoa [41] and are absent from fungi and plants.

Certain structural meiotic proteins were established to be closer to bacterial proteins in their origin, while some others are more similar to the Archaeal proteins [53]. However, the SC proteins generally show a low, if any, similarity with prokaryotic proteins, which does not exceed that between random amino acid sequences and prokaryotic proteins [53]. These findings have led to the conclusion that the SC proteins arose relatively recently in evolution, when primary eukaryotes evolved.

### 3.2. SC Proteins of the Model Organisms with Highest Similarity to Proteins of the Eukaryotes Examined

Yeast Hop1 and* Arabidopsis* ASY1, among all LE components, have similarities primarily with proteins of the algal, moss, fungal, plant, and unicellular animal proteomes. Related proteins were additionally found in the proteomes of highly organized animals. These proteins have the HORMA domain, which structures the chromosomes. The other SC components, such as mouse and fish SC65, SYCP2, and SYCP3, showed significant similarities only with proteins of multicellular organisms.

A consistently high similarity with proteins of even unicellular eukaryotes was observed for FKBP6, which is annotated as an SC component only in mouse. The maximal scores obtained for FKBP6 reached 115 in plants (Table 3), 133 in unicellular eukaryotes (Table 4), 208 in sponges and placozoans (Table 5), and 402 with a molecule size of 327 residues in vertebrates (Table 6). Distant relatives of FKBP6 were found even in prokaryotic proteomes (eubacterial and Archaeal) with maximal scores of 77 and 41, respectively (our data). The SC central space proteins that form transversal filaments (Zip1, C(3)G, ZYP1, SYP-1, and SYCP1) showed a low, but still significant, similarity with proteins from almost all unrelated eukaryotic groups. A quite high similarity was observed with proteins of related groups.

Proteins related to vertebrate SYCE2 were found in the proteomes of Mollusca, Cnidaria (Table 5), and Echinodermata (Table 6). These proteins are possibly not restricted to vertebrates, contrary to the initial assumption [34]. This conclusion agrees with a possible appearance of the SYCE2-like proteins in early Metazoa, as proposed by Fraune et al. [42]. The SC component SC65 occurs not only in Deuterostomia (Table 6), but also, possibly, in Coelenterata, Porifera, and certain Protostomia as well (Table 5). It is remarkable that this protein is annotated in the database for nematodes.

### 3.3. “Exclusive Proteins” in the Proteomes of Certain Eukaryotic Groups

When comparing the SC transversal filament proteins of the model organisms with proteins of Sporozoa, Placozoa, Mollusca, Echinodermata, and Hemichordata, significant similarity of scores was obtained not only for the native proteins, but also for random amino acid sequences generated on the basis of the native proteins by a special program to have the same size and the same amino acid proportion (italicized in Tables 4–6). The same scores were found for the native and random “SC proteins” used as queries. Generally, the scores obtained for random (control) sequences did not exceed 40 and in many cases were below 30. In the above eukaryotic groups, the maximal score reached 70 (e.g., for mouse SYCP1 random analog). The maximal scores obtained with the native proteins for various proteomes were rather low, but still significant: 70–77 for Zip1, 63–70 for C(3)G, 56–83 for ZYP1a, 53–70 for ZYP1b, 67–99 for mouse SYCP1, 44-66 for fish SYCP1, and 50–68 for nematode SYP-1.* E*-values were sufficiently high, ranging, for instance, from *e* ^−9^ down to *e* ^−18^ for the native proteins and from *e* ^−07^ down to *e* ^−12^ for random “proteins” in the case of Placozoa; that is, these results were reliable.

To study the reason of almost equal scores for native and random “proteins”, domain and secondary structure analyses were carried out by us for the proteins occurring in the proteomes of the above eukaryotic groups and displaying a high similarity to the SC transversal filament proteins. The proteins turned to be few; for example, only one “exclusive” protein was found in each of the Mollusca, Hemichordata, and Echinodermata proteomes. The exclusive proteins had large size in all of the proteomes tested, from 3906 residues in Hemichordata to 7710 residues in Placozoa. A similarity to the SC proteins was restricted to their C-terminal regions, which were taken for further analyses. The domain composition of the C-terminal region slightly differed among these exceptional proteins. For instance, myosin 10 and GCC2_GCC3 functional domains were found in Mollusca proteins. The Smc domain, which is responsible for cell division and chromosome segregation, was detected in the Apicomplexa proteins. We found the GCC2_GCC3 and Trichoplein domains in Hemichordata, the Trichoplein domain in Echinodermata, and many various domains, including two Trichoplein and several myosin domains, in Placozoa. The SMC domains, which structure the chromatin and recruit other proteins, are also of particular importance. These domains were found in certain SC proteins (Table 2). It is noteworthy that all of the domains (i.e., the corresponding protein regions) form a distinct *α*-helix, as characteristic of SC transversal filament proteins. The Trichoplein is also of interest, being annotated as a meiosis-specific nuclear structural protein.

We performed a “reverse” BLAST search for two proteins, searching the mouse and* Drosophila* proteomes for proteins similar to the* Trichoplax adhaerens *(Placozoa) exclusive proteins XP_002107637.1 and XP_002111687.1. Apart from one large mouse protein, the mouse and* Drosophila* proteins found were small and showed a similarity to the C-terminal regions of the two* T. adhaerens* proteins. The set included various myosins and, in the case of* Drosophila, *cytoplasmic linker proteins. Both myosins and linker proteins have *α*-helical domains, as the C-terminal domains of the* T. adhaerens* proteins (the program COILS from ExPASy tools was used (http://www.ch.embnet.org/software/COILS_form.html)).

According to bioinformatics criteria, SC transversal filament proteins have much in common with the so-called intermediate proteins, which include proteins of the nuclear lamina, nuclear matrix, and spindle pole body, the myosin heavy chain, and several other proteins. The proteins form an *α*-helical structure, and all *α*-helices have approximately 20% similarity with each other. This is due to repetitive “reference” hydrophobic amino acid residues [13, 22, 32]. The same situation was observed in the case of the above-mentioned exclusive proteins.

Why the proteins found in certain eukaryotic groups are similar not only to the SC transversal filament proteins, but also to random amino acid sequences generated on the basis of the native proteins? Two explanations are possible. First, similar amino acid combinations might occur in the SC proteins and their random “analogs” because repeats are characteristic of the *α*-helices present in the former. Second, errors might occur during computer assembly of the sequenced genomes and corresponding proteomes. Genome sites, coding every revealed protein, may be responsible not for a long, but for two shorter polypeptides, the second one being similar to SC protein. This is why C-terminal regions of the proteins found in our work may belong to SC transversal filament proteins or other intermediate proteins. The hypothesis is based on the fact that *α*-helical proteins occur in many proteomes, while the above phenomenon was only observed for a few proteins from certain eukaryotic proteomes.

## 4. Conclusions and Prospects

Our comparisons enable a conclusion that Hop1, ASY1, and ASY2 are the most universal of all structural SC proteins (Table 7). They have the HORMA functional domain, which recognizes chromatin states and acts as an adaptor that recruits other proteins. We have assumed previously that HORMA domain-containing proteins play a universal role in formation of SCs in higher eukaryotes as well [4]. Similarly, mouse HORMAD1 was recently found to play an essential role in the SC formation and the correct progress of meiosis [54]. Since SC forms on the basis of chromosome axes via protein-protein interactions, it is clear that similar proteins involved in chromosome organization should occur in the proteomes of all eukaryotes capable of meiosis, and this was actually observed in our study.

The LE proteins are possibly the most ancient of all SC proteins. This assumption seems most plausible given that chromosome axes have formed earlier than SC transversal filaments. SYCP2 and SYCP3 replaced Hop1 and Red1 in animals, although HORMA domain-containing proteins are also active in their meiosis [54]. The replacement was possibly associated with complication of genomes. Yet plants with very large genomes have the Hop1 ortholog ASY1. It is possible that SYCP2 and SYCP3 were recruited in vertebrate animals because chromosome-structuring protein complexes had been complicated to include meiosis-specific cohesins and accessory proteins in Vertebrata. SYCP1 and SYCP3 orthologs, which have recently been found in invertebrates [41], display only a low similarity to their vertebrate counterparts (Table 7). The proteins seem to have evolved quite rapidly in parallel with genome complication.

We used mainly the BLAST program with constructing only two phylogenetic trees as examples. It cannot be excluded that protein similarities revealed by using BLAST only cannot provide a basis for phylogenetic inferences [55, 56]. We did not seek particular orthologs for the known SC proteins. Our intention was to find out whether proteins similar to the known SC proteins occur in the proteomes of a particular eukaryotic group. Our results can be used as a basis for a targeted search for orthologs of the SC proteins with the help of phylogenetic trees. The taxa most interesting for such a study were revealed in present investigation and include Chlorophyta, Phaeophyceae, Apicomplexa, Porifera, Placozoa, and Mollusca (Table 7).

The significance of our results usually was high or very high according to the* E*-values obtained which ranged from low significant 3*e* ^−05^ for Zip1 Sc in the proteomes of Parabasalia, Fornicata, and Heterolobosea to very highly significant 8*e* ^−171^ for SC65 Dr in the mammalian proteomes or even 0.0 for SC65 Mm in the amphibian and avian proteomes. The estimates are comparable with or even higher than those specified as essential for correct phylogenetic inferences in the literature, from *e* ^−5^ [57] down to *e* ^−20^ [58].

Thus, we obtained new evidence for the earlier assumption that different proteins whose common feature is the presence of domains with a certain conformation are used to form the SC in different eukaryotic taxa [4, 59]. Here we extended this conclusion from green plants, fungi, and vertebrates to include protozoans and red and brown algae, while Fraune et al. [41] extended it for invertebrates.

The independent evolutionary lineages of multicellular eukaryotes [51] possibly had a common set of basic meiotic proteins, as may be derived from the results of Ramesh et al. [3], and a lineage-specific set of structural proteins, including the SC proteins. Proteins of SC central space are most evolutionarily variable. It implies that different protein-protein interactions can exist to connect two LEs into SC. At the same time, it looks like HORMA domain is the most valuable to assembly the LE itself from different proteins.

Based on our findings, the lack of proteins similar to the SC proteins of the model organisms in Rhodophyta, Euglenophyta, Chrysophyta, Charophyta, Xanthophyta, and Dinoflagellata makes it possible to assume that meiosis in these algae differs from classical meiosis in proceeding without any SC or that unknown new proteins form SCs in these algae. Either alternative is of interest to investigate.

## Figures and Tables

**Table 1:** Eukaryotic SC proteins compared as queries with unidentified proteins from the proteomes of other eukaryotes.

Number	SC protein	Corresponding model organism	Database and protein ID
1	ASY1	Plant *Arabidopsis thaliana* (At)	RefSeq: NP_564896.1
2	ASY2	Plant *Arabidopsis thaliana* (At)	RefSeq: NP_194947.2
3	C(2)M	Insect *Drosophila melanogaster* (Dm)	RefSeq: NP_609788.1
4	C(3)G	Insect *Drosophila melanogaster* (Dm)	GenBank: ACI96726.1
5	CORONA	Insect *Drosophila melanogaster* (Dm)	GenBank: AAF55549.2
6	FKBP6	Mammal *Mus musculus* (Mm)	Swiss-Prot: Q91XW8
7	HIM-3	Nematode *Caenorhabditis elegans* (Ce)	Swiss-Prot: G5EBG0
8	Hop1	Yeast *Saccharomyces cerevisiae* (Sc)	RefSeq: NP_012193.1
9	Hop1	Yeast *Schizosaccharomyces pombe* (Sp)	RefSeq: NP_596448.1
10	Rec10	Yeast *Schizosaccharomyces pombe* (Sp)	RefSeq: NP_594524.1
11	Red1	Yeast *Saccharomyces cerevisiae* (Sc)	RefSeq: NP_013365.1
12	SC65	Fish *Danio rerio* (Dr)	RefSeq: NP_001119910.1
13	SC65	Mammal *Mus musculus* (Mm)	GenBank: CAM23031.1
14	SYCE1-like	Fish* Danio rerio* (Dr)	RefSeq: XP_694355.3
15	SYCE1	Mammal *Mus musculus* (Mm)	RefSeq: NP_001137237.1
16	SYCE2	Fish *Danio rerio* (Dr)	GenBank: AAI33854.1
17	SYCE2	Mammal *Mus musculus* (Mm)	RefSeq: NP_001161718.1
18	SYCE3	Mammal *Mus musculus* (Mm)	RefSeq: NP_001156354.1
19	SYCP1	Fish *Danio rerio* (Dr)	GenBank: AAH45503.1
20	SYCP1	Mammal *Mus musculus* (Mm)	RefSeq: NP_035646.2
21	SYCP2	Fish *Danio rerio* (Dr)	Swiss-Prot: F1QMZ4
22	SYCP2	Mammal *Mus musculus* (Mm)	RefSeq: NP_796165.2
23	SYCP3-like	Fish *Danio rerio* (Dr)	RefSeq: NP_001035440.1
24	SYCP3	Mammal *Mus musculus* (Mm)	RefSeq: NP_035647.2
25	SYP-1	Nematode *Caenorhabditis elegans* (Ce)	Swiss-Prot: G5EGS8
26	SYP-2	Nematode *Caenorhabditis elegans* (Ce)	GenBank: AAC19209.1
27	SYP-3	Nematode *Caenorhabditis elegans* (Ce)	GenBank: CAB03087.2
28	SYP-4	Nematode *Caenorhabditis elegans* (Ce)	RefSeq: NP_491960.1
29	TEX12	Fish *Anoplopoma fimbria* (Af)	GenBank: ACQ58790.1
30	TEX12	Mammal *Mus musculus* (Mm)	GenBank: AAH61081.1
31	Zip1	Yeast *Saccharomyces cerevisiae* (Sc)	RefSeq: NP_010571.1
32	ZYP1a	Plant *Arabidopsis thaliana* (At)	GenBank: AAY46119.1
33	ZYP1b	Plant *Arabidopsis thaliana* (At)	GenBank: AAY46120.1

**Table 2:** Eukaryotic SC proteins, their functional domains, and the total protein size (amino acid residues, aa).

SC central space proteins			LE proteins and other SC proteins		
Protein	Functional domains^a^	Total size, aa	Protein	Functional domains^a^	Total size, aa
Zip1 Sc^b^	Bacterial SMC, Smc, AAA_13	875	Hop1 Sc	HORMA	605

ZYP1a At	Two bacterial SMC domains	871	Red1 Sc	Rec10/Red1	827

ZYP1b At	Two bacterial SMC domains, PRK00409	856	Hop1 Sp, a linear element component	RING finger	528

C(3)G Dm	Two bacterial SMC domains	744	Rec10 Sp, a linear element component	Rec10/Red1	791

CORONA Dm	—	207	ASY1 At	HORMA, SWIRM	596

SYP-1 Ce	Smc	489	ASY2 At	HORMA	1399

SYP-2 Ce	—	213	C(2)M Dm	Rad21_Rec8_N cohesin domain	570

SYP-3 Ce	SGNH_plant_lipase_like	224	HIM-3 Ce	HORMA	291

SYP-4 Ce	—	605	SYCP2 Dr	—	995

SYCP1 Dr	SCP-1	537	SYCP3-like Dr	COR1	240

SYCE1-like Dr	—	206	SC65 Dr, a SC protein	Bacterial rpoC2_cyan	426

SYCE2 Dr	—	187	SYCP2 Mm	Bacterial COG4399	1500

TEX12 Af	—	135	SYCP3 Mm	COR1	254

SYCP1 Mm	SCP-1	993	SC65 Mm, a SC protein	—	443

SYCE1 Mm	Bacterial SMC	329	FKBP6 Mm, peptidyl-prolyl cis-trans isomerase	FKBP_C, TPR	327

SYCE2 Mm	—	177			

SYCE3 Mm	—	88			

TEX12 Mm	—	123			

^a^According to the CDART output.

^b^The model organisms are designated as in Table 1. See protein IDs in Table 1.

The SMC, Smc, SCP-1, COR1, and RAD21 domains are characteristic of structural chromosome proteins. The HORMA domain recognizes the chromatin state and facilitates the interactions with other proteins. PRK00409 is involved in recombination. Cis-trans isomerases catalyze the isomerization of protein molecules having double bonds. The other domains are not related to meiosis.

**Table 3:** SC proteins similar to proteins from the proteomes of algae, mosses, fungi, and green plants.

Eukaryotic taxa	Total proteins in the NCBI database^a^	Proteins of the SC central space		Lateral element proteins and other SC proteins	
		1	2	3	4
		From animals^#^	From plants and fungi^#^	From plants and fungi^#^	From animals^#^
Chlorophyta (green algae and **Viridiplantae**)	156803	SYCP1 Mm (50)	Low similarity	**ASY2 At (117)**, **ASY1 At (163)**, Hop1 Sc (99)	HIM-3 Ce (54), FKBP6 Mm (87)

Phaeophyceae (brown algae and **Stramenopiles)**	27435	SYCP1 Mm (53)	Low similarity	ASY2 At (65), **ASY1 At (124)**, Hop1 Sc (71), Hop1 annotated for *Ectocarpus siliculosus *	FKBP6 Mm (85)

Bryophyta, Anthocerotophyta, and Marchantiophyta (mosses and **Viridiplantae**)	95921	Low similarity	Low similarity	**ASY2 At (183)**, **ASY1 At (278)**, Hop1 Sc (86)	**FKBP6 Mm (104)**

Lycopodiophyta (**Viridiplantae** and Trachaeophyta)	71720	Low similarity	**ZYP1a At (111), ZYP1b At (112)**	**ASY2 At (197), ** **ASY1 At (291),**Hop1 Sc (96)	SYCP2 Mm (60), **FKBP6 Mm (106)**

Euphyllophyta (**Viridiplantae** and Trachaeophyta)	2066225	Low similarity	**ZYP1 annotated for various ** **s** **p** **e** **c** **i** **e** **s** ^b^	**ASY1 annotated for** *Brassica oleracea*;^b^ **Hop1 Sc (100) **	**FKBP6 Mm (115)**

Microsporidia (unicellular lower fungi and **Opisthokonta)**	20596	Low similarity	Low similarity	ASY1 At (53), Hop1 Sp (50), Hop1 Sc (55)	

Blastocladiomycota, Chytridiomycota, Glomeromycota, and Fungi insertae sedis (Zygomycota) (lower fungi and (**Opisthokonta**)	12709	Low similarity	Low similarity	ASY2 At (87), **ASY1 At (126),** **Hop1 Sc (106)**	**FKBP6 Mm (102)**

Ascomycota (**Opisthokonta**)—higher fungi	1819825	SYCP1 Mm (50), SYCP1 Dr (51)	Low similarity^b^	ASY2 At (75), **ASY1 At (100)**, Hop1-like annotated^b^	HIM-3 Ce (50), FKBP6 Mm (77)

Basidiomycota (**Opisthokonta**) and higher fungi	461746	Low similarity	Low similarity	ASY2 At (85), **ASY1 At (123),**Hop1 Sp (74), **Hop1 Sc (141)**	HIM-3 Ce (56), FKBP6 Mm (72)

^a^As of the time of study start (September 2011).

^b^Similarity with cognate proteins is not shown.

Maximal scores are indicated in parentheses. Proteins with high scores (100 and higher) are in bold. The model organisms are designated as in Table 1.

^#^Hereinafter: SC proteins from animals are those of Dm, Ce, Dr, Mm, and from fungi and plants those of Sc, Sp, and At.

**Table 4:** SC proteins with similarities to proteins from the proteomes of unicellular eukaryotes.

Eukaryotic taxa	Total proteins in the NCBI database^a^	Proteins of the SC central space		Lateral element proteins and other SC proteins	
		1	2	3	4
		From animals	From plants and fungi	From plants and fungi	From animals
Parabasalia + Fornicata ++ Heterolobosea (primitive unicellular eukaryotes)	174018	Low similarity	ZYP1a At (50)	**ASY2 At (107)**, **ASY1 At (194)**, **Hop1 Sc (104)**, Hop1 annotated for *Giardia intestinalis* and Hop1-like, for *Trichomonas vaginalis *	HIM-3 Ce (55), SYCP2 Mm (51)

Apicomplexa (sporozoans of the group **Alveolata**)	241035	SYP-1 Ce (57), *SYCP1 Mm (67)^c^*	ZYP1b At (59), *ZYP1a At (60)^c^*	**ASY2 At (107)**, **ASY1 At (148)**, Hop1 Sc (99)	**FKBP6 Mm (133)**

Ciliophora (infusoria, **Alveolata**)	144165	SYCP1 Dr (60)	ZYP1b At (51)	Low similarity	**FKBP6 Mm (114)**

Euglenozoa (euglenic protozoans)	187312	SYCP1 Mm (56)	Low similarity	ASY2 At (91), **ASY1 At (115)**, Hop1 Sc (94)	HIM-3 Ce (52), FKBP6 Mm (78)

Choanoflagellata	30401	C(3)G Dm (57), SYCP1 Mm (65)	ZYP1a At (52), ZYP1b At (61), Zip1 Sc (52)	ASY1 At (78), Hop1 Sc (56)	SYCP2 Mm (53), FKBP6 Mm (76)

^a^As of the time of study (November 2011–November 2012).

^c^These scores (with *E*-value = 5*e* ^−08^) approximate the scores obtained for the random analogs of the SC proteins (*E*-value = 5*e* ^−04^) (italicized).

Other designations are as in Table 3.

**Table 5:** SC proteins with similarities to proteins from the proteomes of multicellular eukaryotes.

Eukaryotic taxa	Total proteins in the NCBI database^a^	Proteins of the SC central space		Lateral element proteins and other SC proteins	
		1	2	3	4
		From animals	From plants and fungi	From plants and fungi	From animals
Porifera and Placozoa (sponges, placozoans, and **Metazoa**)	16445	*SYP-1 Ce (56)^c^*, *C(3)G Dm (70)^c^*, *SYCP1 Dr (66)^c^*, *SYCP1 Mm (88)^c^*	*ZYP1a At (83)^c^*, *ZYP1b At (64)^c^*, *Zip1 Sc (72)^c^*	ASY2 At (76), **ASY1 At (137)**, Hop1 Sc (71)	HIM-3 Ce (56), **SC65 Dr (102), SC65 Mm (110), SYCP3-like Dr (112), SYCP3 Mm (116), ** **FKBP6 Mm (208)**, SCP3(SYCP3)-like annotated for *Amphimedon queenslandica *

Coelenterates: Cnidaria (stingers), Ctenophora (sea walnuts), and **Eumetazoa**	81390	SYCE2 Dr (52), SYCE2 Mm (63)	Low similarity	ASY2 At (78), **ASY1 At (137)**, Hop1 Sc (82)	HIM-3 Ce (68), **SC65 Mm (100), SC65 Dr (105), SYCP3-like Dr (143), SYCP3 Mm (148), ** **FKBP6 Mm (194)**, SYCP3-like annotated for *Hydra magnipapillata *

Platyhelminthes (flat worms, **Eumetazoa**, Bilateria, and Acoelomata)	81455	SYCP1 Mm (53)	Low similarity	**ASY1 At (105),** ASY2 At (62), Hop1 Sc (57)	HIM-3 Ce (68), SYCP2 Mm (53), **FKBP6 Mm (138)**

Nematoda (round worms, **Bilateria**, Coelomata, and Protostomia)	297231	C(3)G Dm (50), SYCP1 Dr (51), SYCP1 Mm (50)^b^	ZYP1a At (52), Zip1 Sc (50)	Hop1 Sc (75), ASY2 At (86), **ASY1 At (124)**	**SC65 Dr (123), SC65 Mm (130)**, FKBP6 Mm (77), SC65 annotated for *Ascaris suum* and HIM-3, for several *Caenorhabditis* species (maximal score = 43)^b^

Mollusca (**Protostomia**)	121831	*C(3)G Dm (68)^c^*, *SYP-1 Ce (68)^c^*, *SYCP1 Dr (64)^c^*, *SYCP1 Mm (99)^c^*, SYCE2 Mm (51)	*ZYP1b At (71)^c^*, *ZYP1a At (81)^c^*, *Zip1 Sc (77)^c^*	ASY1 At (89), Hop1 Sc (55)	SC65 Dr (57), **SYCP3-like Dr (199)**, SC65 Mm (66), SYCP2 Mm (80), **FKBP6 Mm (171), SYCP3 Mm (197)**, SYCP2 and SYCP3 annotated for *Crassostrea gigas *

Chelicerata (Panarthropoda 2 and **Protostomia**)	125005	Low similarity	Low similarity	Low similarity	SC65 Dr (88), SC65 Mm (99), **FKBP6 Mm (179)**

Mandibulata (Panarthropoda 3 and **Protostomia**)	1624768	Low similarity apart from cognate proteins^b^	Low similarity	ASY1 At (63), Hop1 Sp (55), Hop1 Sc (60)	**SC65 Dr (143), FKBP6 Mm (156)**, **SC65 Mm (169)^b^**

^a^As of the time of study (October 2012–April 2013).

^b^Similarity with cognate proteins is not shown.

^c^These scores (with *E*-values ranging from 3*e* ^−10^ to 2*e* ^−20^) approximate the scores obtained for the random analogs of the SC proteins (*E*-values ranging from 2*e* ^−04^ to 1*e* ^−11^) (italicized).

Other designations are as in Table 3.

**Table 6:** SC proteins of the model organisms with similarities to proteins from the proteomes of Deuterostomia.

Eukaryotic taxa	Total proteins in the NCBI database^a^	Proteins of the SC central space		Lateral element proteins and other SC proteins	
		1	2	3	4
		From animals	From plants and fungi	From plants and fungi	From animals
Echinodermata (**Deuterostomia** 1)	41869	SYCE2 Dr (50), SYCE2 Mm (68), *SYCP1 Mm (76)^c^*, C(3)G Dm (63), SYP-1 Ce (59), SYCE2-like annotated for *Strongylocentrotus purpuratus *	ZYP1b At (65), *ZYP1a At (74)^c^*	ASY2 At (69), **ASY1 At (125)**, Hop1 Sc (75)	**FKBP6 Mm (104), SYCP2 Mm (118), SYCP3-like Dr (157), ** **SYCP3 Mm (169), SC65 Dr (176), ** **SC65 Mm (179)**, SCP3-like annotated for *Strongylocentrotus purpuratus *

Hemichordata, Xenoturbellida, and Chaetognatha (**Deuterostomia** 2)	14118	*SYCP1 Mm (69)^c^*, C(3)G Dm (68), SYP-1 Ce (50)	*ZYP1b At (53)^c^*, *ZYP1a At (56)^c^*, Zip1 Sc (70)	ASY2 At (59), **ASY1 At (121)**, Hop1 Sc (85)	FKBP6 Mm (95), **SC65 Dr (177)**, **SC65 Mm (182)**, leprecan-like = SC65-like annotated for *Saccoglossus kowalevskii *

Cephalochordata (**Deuterostomia** 3)	60054	SYCP1 Dr (69^d^), SYCE2 Mm (72), SYCP1 Mm (81^d^), C(3)G Dm (59^d^)	ZYP1a At (68^d^), ZYP1b At (69)	ASY2 At (50^d^), ASY1 At (87^d^), Hop1 Sc (52^d^)	**SYCP2 Mm (115), FKBP6 Mm (125), SC65 Dr (127), SC65 Mm (144), SYCP3-like Dr (193)**, **SYCP3 Mm (194)**

Tunicata (**Deuterostomia**, Chordata)	51435	SYCP1 Dr (55), SYCP1 Mm (57), C(3)G Dm (57)	ZYP1a At (57), ZYP1b At (66)	Low similarity	**SYCP3 Mm (136)**, **SYCP3-like Dr (140)**, **SC65 Mm (150)**, **SC65 Dr (153), FKBP6 Mm (175)**, similar to SCP3-like annotated for *Ciona intestinalis* and leprecan = SC65, for *Molgula tectiformis *

Actinopterygii (**Chordata**, Vertebrata, and Teleostomi)	451775	TEX12 Mm (57), SYCE2 Mm (67), SYCE3 Mm (73), SYCE1 Mm (84),** SYCP1 Mm (320)^b^**, C(3)G Dm (59), SYP-1 Ce (50)	ZYP1a At (57), Zip1 Sc (56)	ASY2 At (70), **ASY1 At (119)**, Hop1 Sc (81)	**SYCP3 Mm (263), FKBP6 Mm (321), SYCP2 Mm (343), SC65 Mm (471)^b^**, HIM-3 Ce (73), SC65-like annotated for various fish species

Amphibia (**Vertebrata**, Teleostomi)	162402	SYCE1-like Dr (50), TEX12 Af (56), TEX12 Mm (72), SYCE2 Mm (84), SYCE1 Mm (93),** SYCE3 Mm (110)**; SYCE1-like, SYCE2-like, TEX12-like annotated for *Xenopus tropicalis *	Low similarity	ASY2 At (82), **ASY1 At (117) **	SYCP2 Dr (50),** SYCP3-like Dr (272), SYCP3 Mm (307), FKBP6 Mm (402), SYCP2 Mm (410), SC65 Dr (464), SC65 Mm (523)**, HIM-3 Ce (63), SYCP2 and SYCP3 annotated for *X. tropicalis,* SYCP2-like and SYCP3, for *X. laevis*; leprecan-like = SC65 precursor, for *X. tropicalis*, *X. laevis *

Sauropsida (**Vertebrata**, Teleostomi)	372873	TEX12 Af (60), SYCE2 Dr (63), SYCE1-like Dr (72), **TEX12 Mm (117), SYCE2 Mm (129), SYCE3 Mm (139), SYCE1 Mm (154), SYCP1 Dr (196), SYCP1 Mm (394)**, C(3)G Dm (52), SYCP1-like, SYCE1-like, SYCE2-like, SYCE3-like, TEX12-like annotated for various bird species	Zip1 Sc (50)	ASY2 At (90), **ASY1 At (124)**	SYCP2 Dr (57),** SYCP3-like Dr (275), SYCP3 Mm (346), FKBP6 Mm (387), SC65 Dr (484), SYCP2 Mm (498), SC65 Mm (574)**, HIM-3 Ce (74), SYCP2-like, SCP3, SC65-like annotated for various bird species

Mammalia (**Vertebrata**, Teleostomi)	2272182	TEX12 Af (64), SYCE2 Dr (68), SYCE1-like Dr (86),** SYCP1 Dr (226)^b^**, SYCP1, SYCE1, SYCE2, SYCE3, TEX12 annotated for various species	Low similarity	ASY2 At (84), **ASY1 At (119)**, Hop1 Sc (78)	**SYCP2 Dr (149)**, **SYCP3-like Dr (275)**, **SC65 Dr (489)^b^**, HIM-3 Ce (79), SYCP2, SYCP3, SC65 annotated for various species

^a^As of the time of study start (January-February 2013).

^b^Similarity with cognate proteins is not shown.

^c^These scores (with *E*-values ranging from 6*e* ^−07^ to 1*e* ^−13^) approximate the scores obtained for the random analogs of the SC proteins (*E*-values ranging from *e* ^−04^ to 4*e* ^−08^) (italicized).

^d^The same proteins (different in different cells of the table) from several proteomes under study showed a maximal similarity to the native protein indicated.

Other designations are as in Table 3.

**Table 7:** SC proteins with highest scores and corresponding eukaryotic taxa.

SC proteins	Maximal scores^a^	Corresponding taxa^b^
Proteins of the SC central space		
SYCP1 Mm^c^	320, 394	Actinopterygii, Sauropsida
SYCE3 Mm	110, 139	Amphibia, Sauropsida
SYCE1, SYCE2, and TEX12 Mm	117–154	Sauropsida
SYCP1 Dr	196, 226	Sauropsida, Mammalia
ZYP1a and ZYP1b At	111-112	Lycopodiophyta

Lateral element proteins and other SC proteins		
ASY1 At	>=100	Algae, Fungi, Parabasalia+, Apicomplexa, Euglenozoa, Porifera, Placozoa, Coelenterates, Platyhelminthes, Nematoda, Deuterostomia 1 and 2, and all Vertebrata
ASY1 At	278, 291	Mosses, Lycopodiophyta
ASY2 At	>=100	Chlorophyta, Parabasalia+, and Apicomplexa
ASY2 At	183, 197	Mosses, Lycopodiophyta
Hop1 Sc	>=100	Euphyllophyta, lower fungi, and Parabasalia+
Hop1 Sc	141	Basidiomycota
SC65 Mm and SC65 Dr	>=100	Porifera, Placozoa, Coelenterates, Nematoda, Mandibulata, and all Deuterostomia
SC65 Mm	523, 574	Amphibia, Sauropsida
SC65 Dr	484, 489	Sauropsida, Mammalia
SYCP3 Mm and SYCP3-like Dr	>=100	Porifera, Placozoa, Coelenterates, Mollusca, Echinodermata, and Cephalochordata, Tunicata, all Vertebrata
SYCP3 Mm	343, 346	Actinopterygii, Sauropsida
SYCP3-like Dr	275	Sauropsida, Mammalia
SYCP2 Mm	>=100	Echinodermata, Cephalochordata, Actinopterygii, and Amphibia
SYCP2 Mm	498	Sauropsida
SYCP2 Dr	149	Mammalia

^a^Similarity with cognate proteins is not shown.

^b^For details see corresponding tables.

^c^The model organisms are designated as in Table 1.
